# Insight into the Wild Origin, Migration and Domestication History of the Fine Flavour Nacional *Theobroma cacao* L. Variety from Ecuador

**DOI:** 10.1371/journal.pone.0048438

**Published:** 2012-11-07

**Authors:** Rey Gaston Loor Solorzano, Olivier Fouet, Arnaud Lemainque, Sylvana Pavek, Michel Boccara, Xavier Argout, Freddy Amores, Brigitte Courtois, Ange Marie Risterucci, Claire Lanaud

**Affiliations:** 1 UMR AGAP, CIRAD, Montpellier, France; 2 EET-Pichilingue, INIAP, Quevedo El Empalme, Ecuador; 3 Centre National de Génotypage, CEA Institut de Génomique, Evry, France; 4 Cocoa Research Unit (CRU), University of the West Indies, St Augustine, Trinidad and Tobago; United States Department of Agriculture, United States of America

## Abstract

Ecuador’s economic history has been closely linked to *Theobroma cacao* L cultivation, and specifically to the native fine flavour Nacional cocoa variety. The original Nacional cocoa trees are presently in danger of extinction due to foreign germplasm introductions. In a previous work, a few non-introgressed Nacional types were identified as potential founders of the modern Ecuadorian cocoa population, but so far their origin could not be formally identified. In order to determine the putative centre of origin of Nacional and trace its domestication history, we used 80 simple sequence repeat (SSR) markers to analyse the relationships between these potential Nacional founders and 169 wild and cultivated cocoa accessions from South and Central America. The highest genetic similarity was observed between the Nacional pool and some wild genotypes from the southern Amazonian region of Ecuador, sampled along the Yacuambi, Nangaritza and Zamora rivers in Zamora Chinchipe province. This result was confirmed by a parentage analysis. Based on our results and on data about pre-Columbian civilization and Spanish colonization history of Ecuador, we determined, for the first time, the possible centre of origin and migration events of the Nacional variety from the Amazonian area until its arrival in the coastal provinces. As large unexplored forest areas still exist in the southern part of the Ecuadorian Amazonian region, our findings could provide clues as to where precious new genetic resources could be collected, and subsequently used to improve the flavour and disease resistance of modern Ecuadorian cocoa varieties.

## Introduction

*Theobroma cacao* L. is the most economically important species of the *Theobroma* genus which is a member of the *Malvaceae* family. It is a diploid perennial species (2n = 2× = 20) with a small genome ranging in size from 411 to 494 Mb [1]. Most of the *T. cacao* accessions are self incompatible due to a gameto-sporophytic incompatibility system [2]–[4]. However, some varieties, domesticated long ago, as the varieties Criollo from central America, Comun from Brazil, or Nacional from Ecuador, and some of their hybrid forms are self compatible.

Traditionnaly, two main genetic groups have been defined to classify cocoa accessions, based on morphological traits and geographical origins [5]: Criollo and Forastero. This classification also reflects the first commonly cultivated cocoa varieties: Criollo, first domesticated in Central America more than 2000 years ago, and a Lower Amazon Forastero variety (Amelonado type) domesticated in Brazil**.** Trinitario, a third group, corresponding to hybrids between Criollo and Forastero, was also recognised. However, the Forastero group also includes many other populations from all the Amazonian and Orinoco regions and presents a high diversity, as revealed by many genetic studies [6], [7], [8]–[16]. A new classification, identifying 10 genetic clusters, was more recently proposed [15].

The domestication of Criollo, originally cultivated by the Mayas in Central America was previously studied [17]. The authors concluded that the genetic base of the ‘ancient’ Criollo variety, cultivated in Central America before foreign cocoa introductions occurred during the 18^th^ century, was very narrow.

Cocoa cultivation in Ecuador is very ancient, dating back to the pre-Columbian age before the Spanish colonization of this territory. Pizarro, during his first voyage in 1526 along the South American coasts (nowadays Ecuador), found evidence of small plantations of an apparently native cocoa tree [18], [19]. It is very likely that the ’Nacional’ Ecuadorian cocoa population existed for several centuries prior to the arrival of the Europeans [20], but its origin in the Ecuadorian coastal region has never been clarified. Two hypotheses have been put forward: according to Allen and Lass [21], the Nacional cocoa variety could have originated from a local wild population, that has nowadays completely disappeared along with the original forest cover of the region, or Nacional could have been introduced in the coastal region from the Amazonian area of Ecuador where wild cocoa is common [22]–[24]. There is no evidence that *T. cacao* and its products played any part in the lives of Ecuadorian coastal inhabitants during the Chorrera phase of the pre-Columbian Valdivia culture (2000 BC), contrary to the extensive amount of information available on this phenomenon with respect to Mesoamerican people, most notably the Mayas and Aztecs. In Ecuador, available information related to the history of Nacional cocoa cultivation is linked with the Spanish colonization history in this country.

Since the time when the first Spanish colonists began deforestation of the Ecuadorian coastal region, a large number of native cocoa trees have been reported [21]–[25], principally along the Guayas basin. Apparently, these colonists began to sow seeds of these native trees approximately 100 years after the discovery of America, and when the native Mesoamerican populations started to decline. The cocoa cultivation areas expanded, and the native Nacional variety rapidly became known worldwide due to its strong floral so-called “Arriba” aroma, exclusively generated by Nacional cocoa beans. The fine flavour quality of chocolate products obtained from Nacional cocoa beans has always been highly appreciated by chocolate manufacturers.

The native Nacional variety was the only one planted in Ecuador until the early 1890s, when foreign germplasm was first introduced in this country. In 1890, due to the quality traits of Nacional cocoa beans, Ecuador had a privileged position in the markets of Hamburg and London [26]. From 1910, foreign germplasm introductions progressively increased due to the appearance of two fungal diseases known as witches’ broom (*Moniliophthora perniciosa*) and frosty pod (*Moniliophthora roreri*), which together devastated the native plantations.

A large genetic admixture between the native variety and foreign germplasm is currently found in modern Ecuadorian cocoa plantations [27]. The fine flavour cocoa aroma has decreased in this hybrid complex, and 25% of the Ecuadorian cocoa production was recently classified as ‘bulk’ cocoa by the International Cocoa Organization (ICCO). There is nowadays increasing demand for fine flavour cocoa, which presently represents 6.8% of the world cocoa production and for which Ecuador remains the main supplier [28].

In a previous work, a few non-introgressed Nacional types were identified within this hybrid population as potential representatives of the native Nacional variety [27]. Here we analyze their relationships with a wide range of wild cocoa genotypes covering a broad geographical range from upper and lower Amazonian regions [21], [22], [29], [30], [31], in order: a) to identify the putative centre of origin of Nacional, and b) to trace the domestication history of the Nacional cocoa variety.

## Materials and Methods

### Plant Material

**Figure 1:**
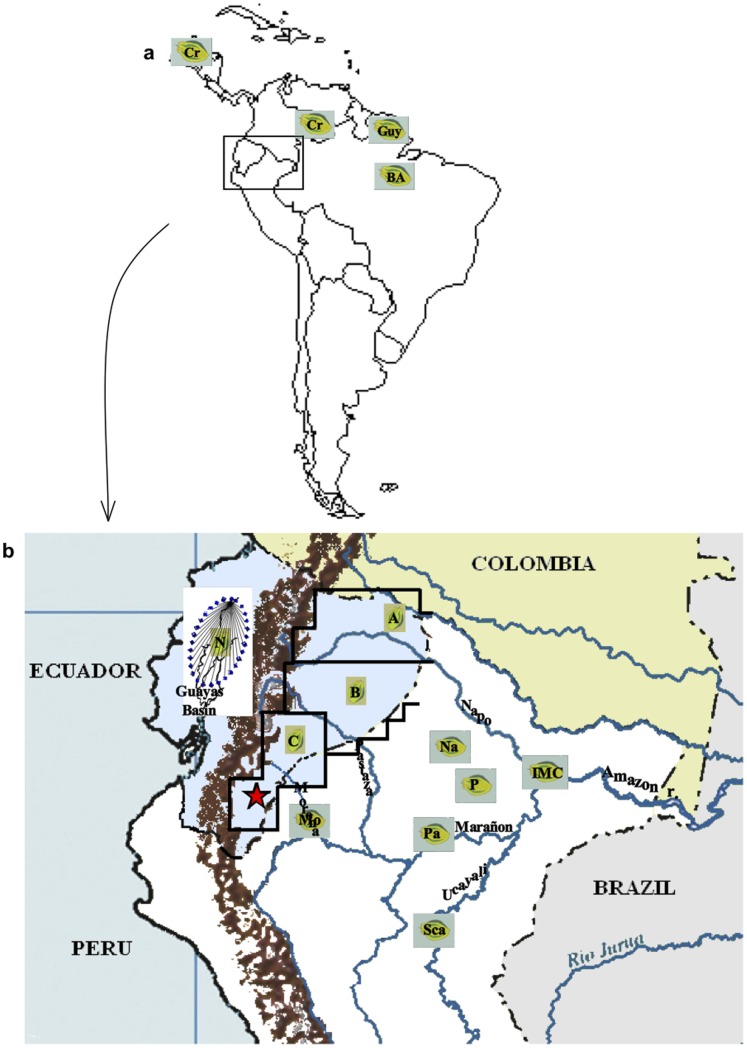
Geographic distribution and locations of the 14 groups of cocoa accessions used in this study. (a) Acessions from Central and South America excepted Ecuador and Peru; and (b) accessions from the Ecuadorian coast and the upper Amazonian region from Ecuador and Peru. The Zone of “Arriba” cocoa is delimited by blue dashes. The locations of Nangaritza and Yacuambi rivers are indicated by a red star. See “material and methods” for explanations of name codes.

A total of 176 individuals from different geographical origins were used for simple sequence repeat (SSR) analyses in this study (Table 1 and Table S1):

- Seven putative ancient Nacional variety individuals cultivated along the Ecuadorian coastal region [27].
- Sixty-five wild genotypes collected in the northern region A (LCT-EENa), central region B (LCT-EENb) and southern region C (LCT-EENc) of the upper Amazonian region of Ecuador (Fig. 1b) [21].
- Sixty-six wild genotypes collected in the upper Amazonian region of Peru (Fig. 1b) [22], [29]:IMC (13 ind.), Nanay (Na, 12 ind.), Parinari (Pa, 13 ind.), Morona (Mo, 8 ind.), Scavina (Sca, 8 ind.), Pound (P, 12 ind.).

- Eleven wild cocoa samples collected in French Guiana (Guy) [30].
- Three cultivated genotypes from the lower Amazonian region of Brazil (BA).
- Twenty-four Criollo (Cr) variety samples collected in Central America, from Venezuela to Mexico [32], were also used in this study.

The germplasm collection and country of origin is reported for each accession in Table S1 and Fig. 1. The wild cocoa accessions from Ecuador and Peru were selected from the CRU living collection on the basis of the geographical area sampled along the Amazonian provinces [21]–[22] (Fig. 1b).

**Table 1:** Origin of the 14 groups of accessions used in this study.

Group name	Code (Fig. 1 and 2)	Origin	Geographical localities or areas	Sample size
Criollo	CR	Central America		22
VEN	VEN	Venezuela	Orinoco (Cuchivero- Padamo)	2
LCT-EENa	LCT-EENa	Ecuador	Amazonia (North region-A)	30
LCT-EENb	LCT-EENb	Ecuador	Amazonia (Centre region-B)	23
LCT-EENc	LCT-EENc	Ecuador	Amazonia (South region-C)	12
Nacional	SNA-B	Ecuador	Coast provinces	7
IMC	IMC	Peru	Iquitos	13
Morona	MO	Peru	Morona river	8
Nanay	NA	Peru	Nanay river	12
Parinari	PA	Peru	Tributaries of Marañon river	13
Pound	P	Peru	Nanay river	12
Scavina	SCA	Peru	Loreto, Ucayali river	8
BA	BA	Brazil		3
GUY	GU	French Guiana	Camopi - Tanpok	11
Total samples				176

Among them Criollo, Nacional and BA correspond to old cultivated varieties. The other groups include cocoa trees native of the mentioned regions.

### Molecular Markers

Eighty SSR markers were chosen for these analyses (Table 2). Seventy four SSRs were isolated from expressed sequence tags (EST) and six from genomic sequences, and mapped [33]–[35]. They are distributed on all of the 10 cocoa chromosomes.

**Table 2:** List of SSR loci (mTcCIRx) and number of alleles per locus identified in this study.

mTcCIR	Nb alleles/locus
1	7
6	11
15	10
26	10
37	11
60	8
292	4
293	5
294	8
297	2
298	6
299	2
302	13
304	6
305	3
308	5
309	4
312	3
316	7
322	15
323	9
324	2
325	7
326	7
327	3
329	10
331	10
333	3
336	7
337	3
339	3
341	2
342	4
344	10
348	3
350	4
352	13
356	4
359	5
361	6
364	2
366	8
369	8
373	5
374	6
375	4
376	6
378	6
379	5
380	4
382	11
383	6
387	4
391	7
392	8
393	8
394	12
396	3
397	2
398	2
400	6
402	3
404	2
405	3
411	7
413	4
417	4
418	2
419	2
420	4
422	13
426	4
427	4
429	8
432	5
435	5
436	3
438	2
446	6
447	4
80	463
	Average 5.78

### DNA Isolation and PCR Amplification

DNA samples were isolated from each individual as described previously [35]. For SSR analyses, PCR amplifications were performed as previously reported [35] and fragments were subsequently detected using a ‘Megabase 1000’ DNA analysis system (Molecular Dynamics/Amersham Life Science).

### Statistical Analysis

Amplified SSR fragments were scored as alleles. The following genetic parameters were calculated for each population using GENETIX software [36]: unbiaised expected heterozygosity [37] (HE), observed heterozygosity (HO), proportion of polymorphic loci at which the frequency of the most common allele does not exceed 95% (P0.95) and 99% (P0.99), and mean number of alleles per locus.

We also estimated allelic richness (Rs) for each population, using a rarefaction approach to correct for unequal sample sizes [38] with the program FSTAT V. 2.9.3 [39].

Wright’s *F*-statistics were estimated according to Weir and Cockerham [40] using the GENETIX software: *F*_IS_, an estimate of heterozygote deficiency or excess, calculated for the whole population sample and for each population, and *F*_ST_, the proportion of variance due to population differenciation, calculated for the whole population sample and between pairs of populations; their significance were assessed using a 1000-fold permutation test.

As most mutations in SSR involve the addition or subtraction of a small number of repeat units, according to the stepwise mutation model population, genetic differentiation was also estimated by *R*_ST_ [41]. *Rho*_ST_, an unbiased version of *R*_ST_ in which allele sizes are transformed to standardize variances, was estimated for pairs of populations using GENEPOP software, WEB version 4.0.10 [42]–[44].

Analyses of genetic distance among pairs of populations [45] were carried out to determine the genetic similarity between the Nacional pool and the wild and cultivated genotypes using GENETIX.

In addition, a dendrogram was constructed using a dissimilarity index (simple matching) and the neighbour-joining (NJ) method with 500 bootstrap replicates. Neighbour-joining cluster analyses were carried out using DARWIN software 5.0 [46].

Finally, a paternity analysis was performed using the software CERVUS, version 2.0 [47], to identify the most likely ancestral population of the Nacional variety. An 80% confidence threshold was used for paternity assignment.

## Results

### Patterns of Genetic Diversity Among Cocoa Accessions

A total of 463 alleles were detected at the 80 analysed SSR loci across all 176 individuals sampled (Table 2). A wide range of allele numbers was generally obtained for each locus (from 2 to 15), with an average of 5.78 alleles per locus. Genetic diversity parameters were evaluated in each group. As shown in Table 3, the mean number of alleles obtained per locus and per accession group corresponding to wild accessions ranged from 1.1 (BA) to 4.45 (LCT-EENa). The allele richness, which takes the sample size into account, varies from 1.05 (CR) to 1.53 (LCT-EENa). For both parameters, the most polymorphic wild cocoa accessions were found in the centre and northern Amazonian regions (A and B) of Ecuador (LCT-EENa and LCT-EENb).

**Table 3:** Genetic diversity parameters revealed by SSR markers within each group analyzed.

Groupname	HE	HO	P(0.95)	P(0.99)	NMA/locus	Allelicrichness	*F*_IS_
Criollo	0.050	0.006	0.150	0.175	1.250	1.050	0.883*
VEN	0.152	0.056	0.225	0.225	1.237	1.152	0.719*
LCT-EENa	0.533	0.317	0.950	0.975	4.450	1.532	0.410*
LCT-EENb	0.485	0.358	0.950	0.962	4.150	1.485	0.266*
LCT-EENc	0.432	0.271	0.787	0.812	2.637	1.432	0.385*
Nacional	0.114	0.094	0.312	0.312	1.375	1.114	0.186*
IMC	0.391	0.479	0.800	0.862	2.700	1.391	−0.236*
Morona	0.321	0.314	0.700	0.700	2.000	1.321	0.034
Nanay	0.234	0.242	0.650	0.750	2.150	1.233	−0.039
Parinari	0.312	0.304	0.637	0.750	2.337	1.312	0.028
Pound	0.399	0.292	0.850	0.900	3.200	1.399	0.277*
Scavina	0.343	0.359	0.750	0.750	2.125	1.343	−0.040
BA	0.068	0.000	0.125	0.125	1.125	1.068	1.000*
Guy	0.114	0.064	0.287	0.325	1.337	1.114	0.457*

HE: Unbiased gene diversity (Nei, 1978) [37]; HO: Observed heterozygosity; Proportion of polymorphic loci at which the frequency of the most common allele does not exceed 95% (0.95) and 99% (0.99); and mean number of alleles per locus (NMA/locus).

Significant *F*_IS_ values were observed in most of the populations with positive values showing an excess of homozygotes and negative values showing an excess of heterozygotes (Table 3). The highest positive values are observed in the BA and Criollo groups, which correspond to self compatible varieties. However, highly homozygous genotypes can also be identified in the other groups such as in the French Guyana, LCT-EEN and Nanay groups (Table S1).

The *F*_IS_ value for the whole sample set and over all loci was 0.2402, with a confidence interval comprised between 0.2155 and 0.2654 after a 1000-fold permutation test.

Genetic differentiation (*F*_ST_) was estimated between pairs of populations (Table 4). Nearly all *F*_ST_ values were significant after permutation tests. Only 3 *F*_ST_ values were not significant: those between the population LCT-EENa from the northern part of the Ecuadorian upper Amazonian region and the two other centre and south regions of the Ecuadorian Amazonia (LCT-EENb and LCT-EENc), and the *F*_ST_ between the Pound and the Nanay populations.

**Table 4:** Coefficients of genetic distance (Nei, 1972) [45] calculated for pair-wise comparisons of the 14 groups of *T. cacao* accessions studied.

	VEN	LCT-EENa	LCT-EENb	LCT-EENc	Nacional	IMC	Morona	Nanay	Parinari	Pound	Scavina	BA	Guy
Criollo	2.141	0.910	0.929	1.079	1.395	1.435	1.293	1.574	1.468	1.461	1.352	2.445	1.611
VEN		0.735	0.892	1.098	1.276	0.385	0.977	0.380	0.402	0.322	1.172	0.233	0.535
LCT-EENa			0.039	0.143	0.492	0.468	0.446	0.619	0.517	0.493	0.631	0.724	0.689
LCT-EENb				0.144	0.582	0.534	0.493	0.715	0.618	0.588	0.652	0.861	0.789
LCT-EENc					0.250	0.632	0.431	0.792	0.718	0.673	0.673	1.110	0.930
Nacional						0.748	0.307	1.017	0.900	0.855	0.779	1.300	1.226
IMC							0.571	0.269	0.312	0.148	0.765	0.416	0.473
Morona								0.906	0.669	0.718	0.615	0.928	0.991
Nanay									0.422	0.051	0.967	0.412	0.586
Parinari										0.303	0.775	0.329	0.304
Pound											0.742	0.347	0.475
Scavina												1.218	0.955
BA													0.557

The *F*_ST_ and *Rho*_ST_ values between cocoa populations were very similar, with slight variations depending on the pair-wise populations (Table 5). Only in the case of the Morona population, its differenciation from the Scavina, VEN and Nacional groups appeared higher when evaluated with the *Rho*_ST_ values compared to the *F*_ST_ values.

**Table 5:** Genetic differenciation between pairs of populations.

Population	Criollo	VEN	LCT-EENa	LCT-EENb	LCT-EENc	Nacional	IMC	Morona	Nanay	Parinari	Pound	Scavina	BA	Guy
Criollo	−	**0.9386**	**0.5804**	**0.6283**	**0.7392**	**0.9107**	**0.7711**	**0.8354**	**0.8552**	**0.8137**	**0.7743**	**0.8279**	**0.9403**	**0.9110**
VEN	0.9144	−	**0.3359**	**0.4112**	**0.4876**	**0.8299**	**0.3545**	**0.5888**	**0.4967**	**0.4134**	**0.2515**	**0.6078**	**0.5133**	**0.7298**
LCT-EENa	0.6004	0.3522	−	***0.0081***	***0.0880***	**0.3582**	**0.2802**	**0.2750**	**0.3824**	**0.3240**	**0.2801**	**0.3310**	**0.3864**	**0.4436**
LCT-EENb	0.6480	0.4269	−0.0018	−	**0.0999**	**0.4303**	**0.3328**	**0.3261**	**0.4462**	**0.3891**	**0.3392**	**0.3714**	**0.4608**	**0.5135**
LCT-EENc	0.7719	0.4362	0.0710	0.0638	−	**0.3191**	**0.3929**	**0.3336**	**0.5165**	**0.4568**	**0.3901**	**0.4149**	**0.5523**	**0.6105**
Nacional	0.9294	0.7884	0.3935	0.4274	0.2812	−	**0.5710**	**0.4727**	**0.7304**	**0.6551**	**0.5820**	**0.6386**	**0.8615**	**0.8442**
IMC	0.7607	0.3062	0.2615	0.2757	0.3451	0.5740	−	**0.4309**	**0.3334**	**0.3204**	**0.1483**	**0.4734**	**0.4279**	**0.5174**
Morona	0.8821	0.6739	0.3154	0.3223	0.3490	0.5671	0.4580	−	**0.6123**	**0.5075**	**0.4557**	**0.4740**	**0.6377**	**0.7147**
Nanay	0.8734	0.5561	0.3501	0.3856	0.4592	0.6960	0.2938	0.6278	−	**0.4703**	***0.0659***	**0.6140**	**0.5670**	**0.6736**
Parinari	0.8483	0.4635	0.2828	0.3341	0.3726	0.5897	0.2909	0.5107	0.4055	−	**0.3031**	**0.5233**	**0.4328**	**0.4887**
Pound	0.7847	0.2825	0.2506	0.2779	0.3197	0.5512	0.1341	0.4693	0.0536	0.2538	−	**0.4549**	**0.3557**	**0.5120**
Scavina	0.8536	0.6008	0.4253	0.4403	0.4528	0.6416	0.4629	0.5525	0.6045	0.5131	0.4194	−	**0.6632**	**0.6929**
BA	0.9389	0.6263	0.4661	0.5238	0.5532	0.8310	0.4772	0.7368	0.6762	0.5198	0.4736	0.6716	−	**0.7752**
GUY	0.9340	0.8182	0.4525	0.5148	0.5821	0.8285	0.4920	0.7632	0.6088	0.3947	0.4547	0.7219	0.8216	−

It was estimated by *F*_ST_ (Weir and Cockerham, 1984) [40], based on variance in allele frequencies (in bold in the table), and *Rho*_ST_, an estimator of *R*_ST_ (Slatkin, 1995) [41] taking the variance in allele sizes into account (values below the diagonal). Non-significant *F*_ST_ values are printed in italic (after a 1000-fold permutation test).

The *F*_ST_ value for the whole multilocus sample set was 0.4979, with a confidence interval comprised between 0.4782 and 0.5192 after a 1000-fold permutation test. The corresponding *Rho*_ST_ value for the whole sample set was 0.530.

**Figure 2:**
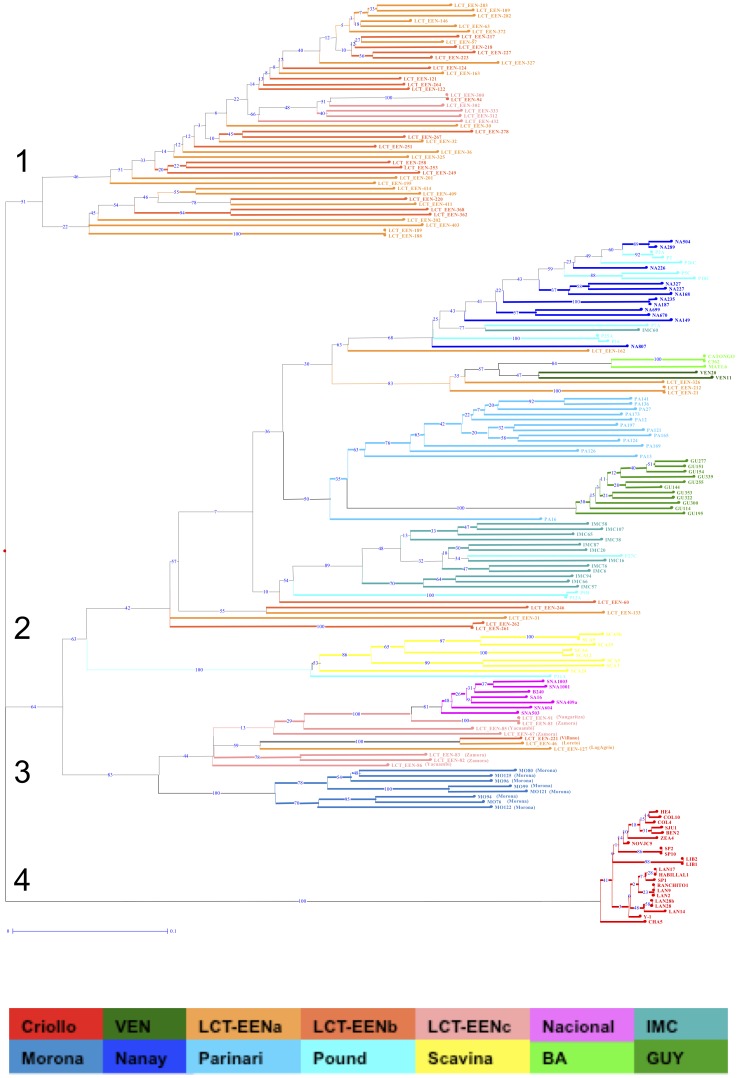
Genetic relationships among the wild and cultivated cocoa accessions. Neighbour-joining tree from a dissimilarity matrix, constructed with 500 bootstraps (the bootstrap values are indicated in blue in each branch), and representing genetic relationships among the whole set of wild and cultivated cocoa accessions analysed in this study. GU (French Guiana), MO (Morona), NA (Nanay), PA (Parinari), P (Pound), SCA (Scavina), SNA (Nacional). The numbers 1 to 4 indicate the cluster numbers reported in the text. Information about the rivers present in the areas where the wild genotypes closest to Nacional were collected (cluster 3), is reported. See Table 1 for code identification.

The neighbour-joining tree (Fig. 2), clustered the majority of cocoa samples according to their geographical origins. Four main clusters were obtained in this analysis (Fig. 2):

Cluster 1 included most of the accessions collected in the northern and central Amazonian regions of Ecuador.
Cluster 2 included most of Peruvian accessions. It was also divided into two subgroups: One subgroup pooled the Scavina accessions; the other subgroup included most of the remaining Peruvian accessions along with wild accessions from French Guiana and Venezuela, and cultivated Lower Amazon samples from Brazil (BA).
Cluster 3 included the Nacional cocoa accessions, the wild cocoa genotypes from the southern part of Ecuadorian Amazonia (region C) and the wild cocoa accessions from Peru collected along the Morona river.
Cluster 4 included all Criollo accessions.

The NJ tree (Fig. 2) highlighted the close relationship between the Nacional pool and the wild cocoa genotypes (LCT-EEN) sampled in the Amazonian region C from Ecuador (Fig. 1b). The accessions sampled in Peru along the Morona river were also genetically close to the Nacional pool; the Morona river region is geographically close to region C of Ecuadorian Amazonia. The largest genetic diversity, expressed by the proportion of polymorphic loci, the mean number of alleles per locus, and the allele richness, was found in the LCT-EEN accessions from Ecuador, and particularly in those originated from regions A and B (Fig. 1b).

### Genetic Distance between the Native Nacional Genotypes and Wild Populations Present in the Amazonian Regions

To refine the previous analysis, coefficients of genetic distance were calculated for pair-wise comparisons of the 14 cocoa groups [45]. A matrix of genetic distance values for all groups is presented in Table 4. The highest genetic distance (2.445) was obtained between the cultivated Criollo and the Lower Amazon group from Brazil (BA), while the most similar populations were LCT-EENa and LCT-EENb, with a genetic distance of 0.039. In the case of the Nacional pool, the lowest genetic distance was noted with the groups from the LCT-EENc region (0.250) and the Morona region (0.307).

Subsequent analyses of genetic distance were carried out to identify the genetic distances between the Nacional pool and wild individuals collected along the neighbouring rivers within each Amazonian region of Ecuador (A, B, C), including all accessions collected in the upper Amazonian region of Peru (data not shown). This new approach allowed us to identify a few LCT-EENc samples (LCT-EEN85, LCT-EEN86 and LCT-EEN91), collected along the adjacent rivers Yacuambi and Nangaritza, as being wild cocoa trees genetically closest to the Nacional pool originally cultivated in the coast region. A key feature of some of the wild cocoa trees from these adjacent rivers is their low heterozygosity level (e.g. 16% for LCT-EEN 91). Note also that the Amazonian region C is geographically close to the Guayas basin (Fig. 1b) where the first Nacional cocoa plantations were established.

### Potential Wild Ancestors of the Nacional Variety

A paternity analysis was performed to identify the most likely representatives of the ancestral population from which the Nacional variety originated. All putative tested parents were from the south Amazonian region C of Ecuador, closest to the Nacional genotypes.

Our results (Table 6) indicated that the LCT-EEN85, LCT-EEN86 and LCT-EEN91 genotypes were the most likely parents of Nacional individuals. These findings were clearly in line with our previous results, suggesting that the native Nacional pool likely descended from wild cocoa trees growing in the vicinity of the Nangaritza and Yacuambi rivers in the southern part of Amazonian region C.

**Table 6:** Most probable ancestors of Nacional individuals.

Individual	Prob. Non-exclusion	First parent	Second parent	
Sa16	6.937×10^−7^	LCT-EEN91		LCT-EEN86
SNA409a	4.072×10^−6^	LCT-EEN86		LCT-EEN91
SNA503	5.139×10^−6^	LCT-EEN91		LCT-EEN85
SNA604	4.001×10^−6^	LCT-EEN91		LCT-EEN85
SNA1001	1.065×10^−6^	LCT-EEN86		LCT-EEN85
SNA1003	6.188×10^−7^	LCT-EEN86		LCT-EEN85
B240	3.893×10^−6^	LCT-EEN91		LCT-EEN86

The two most likely parents of Nacional cocoa individuals were identified with the Cervus 2.0 software parentage analysis. All putative parents tested were from the southern Amazonian region C of Ecuador.

## Discussion

Throughout the history of cocoa cultivation in Ecuador, substantial genetic changes, including reproductive behaviour, have occurred as a consequence of foreign germplasm introductions and genetic admixture associated with natural and human selection. The first study of genetic diversity of modern Ecuadorian *T. cacao* accessions was undertaken by Lerceteau et al. [6], [7]. However, the origin of Nacional cocoa variety, its genetic relationships with wild cocoa trees from Amazonia, and its domestication history had yet to be clarified.

In this study, we used 80 SSR markers to analyse relationships between representatives of the putative founders of the Nacional variety and wild populations from Amazonia in order to identify its wild genetic origin and elucidate the domestication events that occurred before the first Nacional plantations were set up in Ecuador in the Guayas Basin.

The accessions studied here cover most of the geographic origins of known *T. cacao* accessions, except for the Upper Amazonian Brazilian accessions, which were not very represented in this study, but for which no specific cluster was identified in the last proposed classification [15]. In our study, the diversity was first structured in four main groups, corresponding to different geographic origins, and with subdivisions within some of these four main groups:

- The first group (cluster 1) included mostly accessions from the northern and central regions of Ecuadorian Amazonia (regions A and B), but without any structuring between them. This is in agreement with the very low genetic distance (0.039) and low genetic differentiation (*F*_ST_ of 0.0081 and *Rho*_ST_ of −0.0018) between these 2 groups.
- The second group (cluster 2) included most of the Peruvian accessions, i.e. mainly those collected in the northern region of Peru, associated with wild accessions collected in French Guiana and in the Orinoco region of Venezuela, and with some accessions from Ecuador. The cultivated genotypes from Lower Amazonia in Brazil were also included in this group.

The Upper Amazonian region has always been considered as the primary centre of origin and diversity of *T. cacao* L. species [5], [48]. The high genetic similarity observed between some wild cocoa accessions from the Upper Amazonian region (Parinari or Pound) and the group from French Guiana, suggests that during *T. cacao* evolution the extension of cocoa populations towards the eastern part of South America and until the French Guyana region, could have started from this Parinari Upper Amazonian region.

- The third group (cluster 3) included most of the accessions from the southern Amazonian region of Ecuador, the accessions collected in Peru along the Morona river, close to this southern Ecuadorian region, and the Nacional accessions. In this group, a close genetic relationship was found among some wild accessions from the southern part of Ecuadorian Amazonia (region C), and the Nacional pool from the coastal region. These results were supported by the genetic distance assessment, as well as by a parentage analysis. Our results suggest that the Amazonian region located in the Province of Zamora Chinchipe could be the centre of domestication of the Nacional variety at the origin of the first Nacional cocoa plantation that was set up on the Guayas riverbanks. Later, from this latter plantation, cocoa cultivation could have expanded along the Guayas tributary rivers (upper waters), which is now the ‘Arriba’ cocoa production region in the provinces of Guayas, Los Ríos and Manabí (Fig. 1b).
- The fourth group (cluster 4) included all Criollo accessions collected from Mexico to Venezuela, reflecting its narrow genetic base. In a previous study, some accessions from Colombia (EBC) were identified as being the closest to Criollo [17]. It has been suggested that Colombia is the centre of origin of Criollo. In our study, we did not have access to EBC Colombian accessions, but the higher genetic similarity and lower genetic differenciation (FST and RhoST values) observed between Criollo and accessions from the northern Amazonian region of Ecuador showed the same geographic trend as previously noted [17].

This migration and domestication hypothesis is supported by the geographical proximity between the first cocoa plantation set up along the Guayas riverbanks [26] and the wild cocoa collected along the Nangaritza, Yacuambi and Zamora riverbanks in the province of Zamora Chinchipe (Fig. 1b, region C). The morphological similarity between the Nacional variety and the native cocoa populations located in the Ecuadorian jungle (fruit and seed) close to the Amazonian cities of Archidona and Macas in region C, and already reported [49], further supports this hypothesis.

Inbreeding coefficients (*F*_IS_) demonstrate a clear and significant excess of homozygotes in some groups. It is the case for the old domesticated and self compatible varieties Criollo and BA varieties, but also for some wild genotypes such as those of the VEN, GUY and LCT-EEN groups. Highly homozygous wild genotypes were identified in each region sampled. This latter result suggests that self compatible alleles already existed in these wild populations and that natural selection could have already eliminated part of the genetic burden due to inbreeding, thus facilitating the process of domestication of some wild populations.

Most representatives of the native Nacional variety have a high level of homozygosity, probably associated with their self compatibility [50]–[52]. The question is whether the domestication process has fixed and selected, by selfing, highly homozygous genotypes, or whether natural selection, leading to increased homozygosity, was already under way in wild populations before this domestication event. A common domestication feature is the reduction of genetic diversity in crops relative to wild progenitors. The severity of genetic loss ascribed to bottleneck effects varies greatly among crop species. Some authors indicated that this reduction results from two major forces [53]: first, most domestication events are thought to have involved initial populations of small size (relative to wild ancestors) that contained a narrow level of genetic diversity; the second factor to have an impact on crop genomes is the selection in favour of agronomic traits that distinguish crops from their ancestors. Humans usually apply selection pressure on the ancestral gene pool to select for favourable traits, thus increasing or fixing favourable alleles at genes controlling these traits.

The particular characteristics of some cocoa varieties could have facilitated the selection and migration of specific cocoa materials. From this standpoint, the specific aromatic flavour of chocolate produced from Nacional cocoa beans, which can already be detected on the bean pulp, even without a fermentation step, could have been one of the criteria used by the primitive human communities to choose the cocoa mother tree for further seed sowing. Indeed, it is possible that travelling merchants transporting cocoa pods along the roads used the fresh pulp only for their own refreshment and nutrition, but without consuming the cocoa beans, thereby introducing the cocoa tree into a new environment [54]. An extensive flavour aroma study in wild genotypes combined with an evolutionary analysis of this trait variation could help to identify candidate genes responsible for flavour in the aromatic Nacional variety.

Many crop species have been domesticated for thousands of years. Archeological evidence, based mainly on the presence of theobromine in pottery residue, revealed that cocoa was used in the early formative period in Mesoamerica, dating back to 600 BC until 1900 BC [55]–[58].

Cocoa domestication seems to have occurred much more recently in most of the other cultivating countries, despite the fact that wild cocoa populations exist in their areas, but no archeological evidence has been found so far in these countries. In Ecuador, information on the domestication of its native cocoa plantations dates back only four centuries.

There is no evidence of human dispersal of this species before the first cocoa plantations were planted by the Spanish in the Ecuadorian coastal region. Knowledge of the dispersal mechanism involved in the long-distance migration process is thus essential to explain our results. Several hypotheses have been put forward to explain the cocoa migration process, such as: the transport of fruits or seeds by birds, animals or humans, but so far none of these has been formally confirmed. It was suggested that the Nacional cocoa variety arrived via the old Inca roads and was planted by the native people of that time along the coastal regions [49]. However, archaeological evidence suggests that these old roads were not built by the Incas but rather by pre-Columbian native people, who inhabited the coastal, Andean and Amazonian regions of Ecuador [59] thousands of years before the arrival of the Incas in the Ecuadorian regions.

Recently, a ceremonial formative site was discovered on the eastern slopes of the Andes in the southern part of Ecuadorian Amazonia, and dating back to the 3rd millennium BC. The cultural remains showed a high degree of development of these societies, with ceramics and marine shells (*Spondyle* and *Strombus*), which is evidence that commercial exchanges with coastal people occured in this region [60], [61]. This site is located in the province of Zamora Chinchipe (region C), where the putative ancestors of the Nacional variety were identified. Archaeological evidence also showed that these exchanges went as far as a place presently known as La Cueva de los Tayos, which is located in the province of Morona Santiago [59].

Unfortunately, contrary to the Mayas who used hieroglyphs and represented cocoa, the more primitive people that inhabited Ecuador did not use writing symbols, so there is little evidence of cocoa domestication and use before Spanish arrival. However, archaeological evidence about contacts and exchanges of products between pre-Columbian peoples from coastal, sierran and Amazonian regions of Ecuador, dating back 3000 years BC [60], [62], could provide further explanations on the origin of cocoa trees in the Ecuadorian coastal region.

From our results, future cocoa expeditions should be carried out to confirm that the southern Amazonian area of Ecuador, could be the putative centre of origin of the Nacional variety, and to collect new germplasm with the same Nacional flavour specificities adapted for fine flavour cocoa improvement in Ecuador.

## Supporting Information

Table S1 **Origin, collection, % heterozygosity and status of the 176** ***T. cacao*** **accessions analysed in the present study.** CATIE: Centro Agronomico Tropical de Investigación y Ensenanza (Costa Rica); CIC: Centro de Investigación Caribia (Colombia); CIRAD/Mpl: Centre de Coopération internationale en recherche agronomique pour le développement (France)/Montpellier Centre; CNRA: Centre National de Recherches Agronomiques (Côte d’Ivoire); CRU: Cocoa research Unit – University of the West Indies (Trinidad and Tobago); EET-P: Estación Experimental Tropical-Pichilingue (Ecuador); FONAIAP: Fondo Nacional de Investigaciones Agropecuarias (Venezuela); INTA: Instituto Nicaraguense de Tecnologìa Agropecuario (Nicaragua); INIFAP: Instituto Nacional de Investigaciones Forestales. Agrícolas y Pecuarias (Mexico). The % of heterozygosity was evaluated by 80 SSR (as reported in Table 2).
(DOCX)
