# Correction: Insight into the Wild Origin, Migration and Domestication History of the Fine Flavour Nacional *Theobroma cacao* L. Variety from Ecuador

**DOI:** 10.1371/annotation/2357f0f1-7dc3-4781-afb0-29a8ce56b3f0

**Published:** 2013-02-08

**Authors:** Rey Gaston Loor Solorzano, Olivier Fouet, Arnaud Lemainque, Sylvana Pavek, Michel Boccara, Xavier Argout, Freddy Amores, Brigitte Courtois, Ange Marie Risterucci, Claire Lanaud

There was an error in the Funding Information. The correct Funding Information is as follows:

The study was funded by the United States Department of State (U.S. Foreign Ministry); the U.S. Embassy, Quito; the U.S. Department of Agriculture (USDA-ARS); the Instituto Nacional Autonomo de Investigaciones Agropecuarias (INIAP-Ecuador); and the Centre de Coopération Internationale en Recherche Agronomique pour le Développement (CIRAD-France). 

