# Supplementary material for: Insight into the Wild Origin, Migration and Domestication History of the Fine Flavour Nacional Theobroma cacao L. Variety from Ecuador
Source: PLoS One. 2012 Nov 7;7(11):e48438. doi: 10.1371/journal.pone.0048438 (PMC3492346; doi:10.1371/journal.pone.0048438)
Supplement: Table S1 — Origin, collection, % heterozygosity and status of the 176 T. cacao accessions analysed in the present study. CATIE: Centro Agronomico Tropical de Investigación y Ensenanza (Costa Rica); CIC: Centro de Investigación Caribia (Colombia); CIRAD/Mpl: Centre de Coopération internationale en recherche agronomique pour le développement (France)/Montpellier Centre; CNRA: Centre National de Recherches Agronomiques (Côte d’Ivoire); CRU: Cocoa research Unit – University of the West Indies (Trinidad and Tobago); EET-P: Estación Experimental Tropical-Pichilingue (Ecuador); FONAIAP: Fondo Nacional de Investigaciones Agropecuarias (Venezuela); INTA: Instituto Nicaraguense de Tecnologìa Agropecuario (Nicaragua); INIFAP: Instituto Nacional de Investigaciones Forestales. Agrícolas y Pecuarias (Mexico). The % of heterozygosity was evaluated by 80 SSR (as reported in Table 2). (DOCX) [file pone.0048438.s001.docx]

**Supplementary Table S1**

| Accession  name | Group name | Country of origin  or domestication | River or site of collect | Status | Collection | % heterozy-gosity |
| --- | --- | --- | --- | --- | --- | --- |
|  |  |  |  |  |  |  |
| B240 | Nacional | Ecuador | Coast provinces | Cultivated | EET-P | 25.0 |
| BEN2 | Criollo | Venezuela | Merida | Cultivated | FONAIAP | 0.0 |
| CATONGO | BA | Brazil | Uruçuca | Cultivated | CRU | 0.0 |
| CHA5 | Criollo | Mexico | Chiapas | Cultivated | - | 4.3 |
| COL10 | Criollo | Colombia | Santa Marta | Cultivated | CIC | 0.0 |
| COL4 | Criollo | Colombia | Santa Marta | Cultivated | CIC | 0.0 |
| GU114-P | Guy | French Guiana | Tanpok | wild | CRU | 8.9 |
| GU144 | Guy | French Guiana | Camopi | wild | CIRAD/Mpl | 15.4 |
| GU151-F | Guy | French Guiana | Camopi | wild | CRU | 2.6 |
| GU154 | Guy | French Guiana | Camopi | wild | CIRAD/Mpl | 6.5 |
| GU195-P | Guy | French Guiana | Camopi | wild | CRU | 2.7 |
| GU255-P | Guy | French Guiana | Camopi | wild | CRU | 5.1 |
| GU277-G | Guy | French Guiana | Camopi | wild | CRU | 2.7 |
| GU300-P | Guy | French Guiana | Camopi | wild | CRU | 5.2 |
| GU322-P | Guy | French Guiana | Camopi | wild | CRU | 11.8 |
| GU339-M | Guy | French Guiana | Camopi | wild | CRU | 0.0 |
| GU353-P | Guy | French Guiana | Camopi | wild | CRU | 9.0 |
| HABILLAL1 | Criollo | Mexico | Michoacan | Cultivated | - | 0.0 |
| HE4 | Criollo | Venezuela | Tachira | Cultivated | - | 0.0 |
| IFC362 | BA | Brazil | Uruçuca | Cultivated | CNRA | 0.0 |
| IMC107 | IMC | Peru | Iquitos | wild | CRU | 52.0 |
| IMC16 | IMC | Peru | Iquitos | wild | CRU | 50.6 |
| IMC20 | IMC | Peru | Iquitos | wild | CRU | 48.1 |
| IMC38 | IMC | Peru | Iquitos | wild | CRU | 37.5 |
| IMC57 | IMC | Peru | Iquitos | wild | CRU | 60.3 |
| IMC58 | IMC | Peru | Iquitos | wild | CRU | 41.3 |
| IMC6 | IMC | Peru | Iquitos | wild | CRU | 46.8 |
| IMC60 | IMC | Peru | Iquitos | wild | CRU | 45.3 |
| IMC65 | IMC | Peru | Iquitos | wild | CRU | 36.8 |
| IMC66 | IMC | Peru | Iquitos | wild | CRU | 48.7 |
| IMC76 | IMC | Peru | Iquitos | wild | CRU | 53.8 |
| IMC87 | IMC | Peru | Iquitos | wild | CRU | 45.3 |
| IMC94 | IMC | Peru | Iquitos | wild | CRU | 49.4 |
| LAN14 | Criollo | Mexico | Chiapas | Cultivated | - | 1.3 |
| LAN17 | Criollo | Mexico | Chiapas | Cultivated | - | 0.0 |
| LAN2 | Criollo | Mexico | Chiapas | Cultivated | - | 0.0 |
| LAN28 | Criollo | Mexico | Chiapas | Cultivated | - | 0.0 |
| LAN28b | Criollo | Mexico | Chiapas | Cultivated | - | 0.0 |
| LAN9 | Criollo | Mexico | Chiapas | Cultivated | - | 0.0 |
| LCT-EEN-109 | LCT-EENa | Ecuador | Napo/Coca | wild | CRU | 21.3 |
| LCT-EEN-121 | LCT-EENb | Ecuador | Bobonaza | wild | CRU | 16.2 |
| LCT-EEN-122 | LCT-EENb | Ecuador | Bobonaza | wild | CRU | 23.5 |
| LCT-EEN-124 | LCT-EENb | Ecuador | Bobonaza | wild | CRU | 16.9 |
| LCT-EEN-127 | LCT-EENa | Ecuador | Coca | wild | CRU | 22.1 |
| LCT-EEN-133 | LCT-EENa | Ecuador | Coca | wild | CRU | 74.6 |
| LCT-EEN-146 | LCT-EENa | Ecuador | Coca | wild | CRU | 23.4 |
| LCT-EEN-162 | LCT-EENa | Ecuador | Napo/Coca | wild | CRU | 38.4 |
| LCT-EEN-163 | LCT-EENa | Ecuador | Napo/Coca | wild | CRU | 27.1 |
| LCT-EEN-188 | LCT-EENa | Ecuador | Putumayo | wild | CRU | 42.7 |
| LCT-EEN-189 | LCT-EENa | Ecuador | Putumayo | wild | CRU | 40.5 |
| LCT-EEN-195 | LCT-EENa | Ecuador | Putumayo | wild | CRU | 27.3 |
| LCT-EEN-201 | LCT-EENa | Ecuador | San Miguel | wild | CRU | 37.9 |
| LCT-EEN-202 | LCT-EENa | Ecuador | San Miguel | wild | CRU | 66.2 |
| LCT-EEN-21 | LCT-EENa | Ecuador | Napo | wild | CRU | 17.1 |
| LCT-EEN-212 | LCT-EENa | Ecuador | Napo | wild | CRU | 15.5 |
| LCT-EEN-217 | LCT-EENb | Ecuador | Villano | wild | CRU | 9.9 |
| LCT-EEN-218 | LCT-EENb | Ecuador | Villano | wild | CRU | 17.6 |
| LCT-EEN-220 | LCT-EENb | Ecuador | Villano | wild | CRU | 36.6 |
| LCT-EEN-221 | LCT-EENb | Ecuador | Villano | wild | CRU | 56.6 |
| LCT-EEN-223 | LCT-EENb | Ecuador | Villano | wild | CRU | 14.9 |
| LCT-EEN-227 | LCT-EENb | Ecuador | Villano | wild | CRU | 16.7 |
| LCT-EEN-246 | LCT-EENb | Ecuador | Villano | wild | CRU | 56.6 |
| LCT-EEN-249 | LCT-EENb | Ecuador | Curaray | wild | CRU | 42.3 |
| LCT-EEN-251 | LCT-EENb | Ecuador | Curaray | wild | CRU | 29.9 |
| LCT-EEN-253 | LCT-EENb | Ecuador | Curaray | wild | CRU | 33.3 |
| LCT-EEN-258 | LCT-EENb | Ecuador | Curaray | wild | CRU | 47.1 |
| LCT-EEN-261 | LCT-EENb | Ecuador | Curaray | wild | CRU | 71.0 |
| LCT-EEN-262 | LCT-EENb | Ecuador | Curaray | wild | CRU | 76.0 |
| LCT-EEN-264 | LCT-EENb | Ecuador | Curaray | wild | CRU | 29.7 |
| LCT-EEN-267 | LCT-EENb | Ecuador | Curaray | wild | CRU | 29.2 |
| LCT-EEN-278 | LCT-EENb | Ecuador | Curaray | wild | CRU | 29.9 |
| LCT-EEN-282 | LCT-EENa | Ecuador | Coca | wild | CRU | 11.8 |
| LCT-EEN-283 | LCT-EENa | Ecuador | Coca | wild | CRU | 15.6 |
| LCT-EEN-30 | LCT-EENa | Ecuador | Capocuy | wild | CRU | 20.8 |
| LCT-EEN-300 | LCT-EENc | Ecuador | Upano | wild | CRU | 19.2 |
| LCT-EEN-302 | LCT-EENc | Ecuador | Upano | wild | CRU | 16.9 |
| LCT-EEN-31 | LCT-EENa | Ecuador | Indillana | wild | CRU | 60.3 |
| LCT-EEN-312 | LCT-EENc | Ecuador | Upano | wild | CRU | 13.8 |
| LCT-EEN-32 | LCT-EENa | Ecuador | Indillana | wild | CRU | 28.8 |
| LCT-EEN-325 | LCT-EENa | Ecuador | Napo | wild | CRU | 43.8 |
| LCT-EEN-326 | LCT-EENa | Ecuador | Napo | wild | CRU | 31.2 |
| LCT-EEN-327 | LCT-EENa | Ecuador | San Miguel | wild | CRU | 2.6 |
| LCT-EEN-333 | LCT-EENc | Ecuador | Upano | wild | CRU | 11.4 |
| LCT-EEN-36 | LCT-EENa | Ecuador | Anangu | wild | CRU | 22.9 |
| LCT-EEN-362 | LCT-EENb | Ecuador | Conanbo | wild | CRU | 31.9 |
| LCT-EEN-368 | LCT-EENb | Ecuador | Conanbo | wild | CRU | 43.7 |
| LCT-EEN-372 | LCT-EENa | Ecuador | Napo | wild | CRU | 22.7 |
| LCT-EEN-403 | LCT-EENa | Ecuador | Yasuni | wild | CRU | 46.5 |
| LCT-EEN-409 | LCT-EENa | Ecuador | Napo | wild | CRU | 38.4 |
| LCT-EEN-411 | LCT-EENa | Ecuador | Aguarico | wild | CRU | 39.7 |
| LCT-EEN-414 | LCT-EENa | Ecuador | Aguarico | wild | CRU | 41.5 |
| LCT-EEN-432 | LCT-EENc | Ecuador | Santiago | wild | CRU | 22.7 |
| LCT-EEN-46 | LCT-EENa | Ecuador | Napo/Loreto | wild | CRU | 39.7 |
| LCT-EEN-57 | LCT-EENa | Ecuador | Napo/Loreto | wild | CRU | 18.9 |
| LCT-EEN-60 | LCT-EENb | Ecuador | Puyo | wild | CRU | 53.9 |
| LCT-EEN-63 | LCT-EENa | Ecuador | San Miguel | wild | CRU | 2.7 |
| LCT-EEN-67 | LCT-EENc | Ecuador | Zamora | wild | CRU | 26.5 |
| LCT-EEN-81 | LCT-EENc | Ecuador | Zamora | wild | CRU | 19.2 |
| LCT-EEN-82 | LCT-EENc | Ecuador | Zamora | wild | CRU | 54.8 |
| LCT-EEN-83 | LCT-EENc | Ecuador | Zamora | wild | CRU | 51.5 |
| LCT-EEN-85 | LCT-EENc | Ecuador | Yacuambi | wild | CRU | 30.6 |
| LCT-EEN-86 | LCT-EENc | Ecuador | Yacuambi | wild | CRU | 32.0 |
| LCT-EEN-91 | LCT-EENc | Ecuador | Nangaritza | wild | CRU | 16.0 |
| LCT-EEN-94 | LCT-EENb | Ecuador | Curaray | wild | CRU | 21.7 |
| LIB1 | Criollo | Nicaragua | INTA | Cultivated | INTA | 0.0 |
| LIB2 | Criollo | Nicaragua | INTA | Cultivated | INTA | 0.0 |
| MAT1.6 | BA | Costa Rica | Matina selection | Cultivated | CATIE | 0 |
| MO121 | Morona | Peru | Morona | wild | CRU | 31.6 |
| MO122 | Morona | Peru | Morona | wild | CRU | 31.6 |
| MO125 | Morona | Peru | Morona | wild | CRU | 35.9 |
| MO76 | Morona | Peru | Morona | wild | CRU | 21.1 |
| MO80 | Morona | Peru | Morona | wild | CRU | 31.9 |
| MO94 | Morona | Peru | Morona | wild | CRU | 23.1 |
| MO96 | Morona | Peru | Morona | wild | CRU | 32.4 |
| MO99 | Morona | Peru | Morona | wild | CRU | 33.3 |
| NA149 | Nanay | Peru | Nanay | wild | CRU | 35.1 |
| NA168 | Nanay | Peru | Nanay | wild | CRU | 19.0 |
| NA187 | Nanay | Peru | Nanay | wild | CRU | 22.4 |
| NA226 | Nanay | Peru | Nanay | wild | CRU | 14.9 |
| NA227 | Nanay | Peru | Nanay | wild | CRU | 28.2 |
| NA235 | Nanay | Peru | Nanay | wild | CRU | 25.0 |
| NA289 | Nanay | Peru | Nanay | wild | CRU | 0.0 |
| NA327 | Nanay | Peru | Nanay | wild | CRU | 25.3 |
| NA504 | Nanay | Peru | Nanay | wild | CRU | 1.3 |
| NA670 | Nanay | Peru | Nanay | wild | CRU | 36.0 |
| NA699 | Nanay | Peru | Nanay | wild | CRU | 35.1 |
| NA807 | Nanay | Peru | Nanay | wild | CRU | 48.0 |
| NOVJC5 | Criollo | Venezuela | Tachira | Cultivated | FONAIAP | 1.4 |
| P10C | Pound | Peru | Nanay | wild | CRU | 0.0 |
| P12A | Pound | Peru | Nanay | wild | CRU | 48.6 |
| P16 | Pound | Peru | Nanay | wild | CRU | 34.7 |
| P2 | Pound | Peru | Nanay | wild | CRU | 10.5 |
| P25A | Pound | Peru | Napo | wild | CRU | 35.4 |
| P26C | Pound | Peru | Napo | wild | CRU | 14.1 |
| P27C | Pound | Peru | Nanay | wild | CRU | 56.0 |
| P2A | Pound | Peru | Nanay | wild | CRU | 11.0 |
| P31A | Pound | Peru | Contamana | wild | CRU | 32.9 |
| P5C | Pound | Peru | Nanay | wild | CRU | 7.6 |
| P7A | Pound | Peru | Nanay | wild | CRU | 37.2 |
| P9B | Pound | Peru | Nanay | wild | CRU | 52.0 |
| PA12 | Parinari | Peru | Parinari | wild | CRU | 29.2 |
| PA121 | Parinari | Peru | Parinari | wild | CRU | 28.6 |
| PA124 | Parinari | Peru | Parinari | wild | CRU | 27.4 |
| PA126 | Parinari | Peru | Parinari | wild | CRU | 28.8 |
| PA13 | Parinari | Peru | Parinari | wild | CRU | 47.2 |
| PA136 | Parinari | Peru | Parinari | wild | CRU | 23.1 |
| PA141 | Parinari | Peru | Parinari | wild | CRU | 20.0 |
| PA16 | Parinari | Peru | Parinari | wild | CRU | 18.8 |
| PA165 | Parinari | Peru | Parinari | wild | CRU | 31.9 |
| PA169 | Parinari | Peru | Parinari | wild | CRU | 32.1 |
| PA173 | Parinari | Peru | Parinari | wild | CRU | 32.9 |
| PA197 | Parinari | Peru | Parinari | wild | CRU | 23.5 |
| PA27 | Parinari | Peru | Parinari | wild | CRU | 34.2 |
| RANCHITO1 | Criollo | Mexico | Michoacan | Cultivated | INIFAP | 0.0 |
| SA16 | Nacional | Ecuador | Coast provinces | Cultivated | EET-P | 10 |
| SCA12 | Scavina | Peru | Contamana | wild | CRU | 39.7 |
| SCA19 | Scavina | Peru | Contamana | wild | CRU | 38.0 |
| SCA24 | Scavina | Peru | Contamana | wild | CRU | 34.7 |
| SCA3 | Scavina | Peru | Contamana | wild | CRU | 36.5 |
| SCA5 | Scavina | Peru | Contamana | wild | CRU | 33.8 |
| SCA6 | Scavina | Peru | Contamana | wild | CRU | 38.8 |
| SCA9 | Scavina | Peru | Contamana | wild | CRU | 33.8 |
| SCA9b | Scavina | Peru | Contamana | wild | CRU | 29.5 |
| SJU1 | Criollo | Venezuela | Guasare | Cultivated | FONAIAP | 0.0 |
| SNA1001 | Nacional | Ecuador | Coast provinces | Cultivated | EET-P | 10 |
| SNA1003 | Nacional | Ecuador | Coast provinces | Cultivated | EET-P | 10 |
| SNA409 | Nacional | Ecuador | Coast provinces | Cultivated | EET-P | 50 |
| SNA503 | Nacional | Ecuador | Coast provinces | Cultivated | EET-P | 22.5 |
| SNA604 | Nacional | Ecuador | Coast provinces | Cultivated | EET-P | 15 |
| SP1 | Criollo | Venezuela | Zulia | Cultivated | FONAIAP | 0.0 |
| SP10 | Criollo | Venezuela | Zulia | Cultivated | FONAIAP | 0.0 |
| SP2 | Criollo | Venezuela | Zulia | Cultivated | FONAIAP | 0.0 |
| VEN11 | VEN | Venezuela | Orinoco | wild | CIRAD/Mpl | 9.9 |
| VEN20 | VEN | Venezuela | Orinoco | wild | CIRAD/Mpl | 0.0 |
| Y-1 | Criollo | Mexico | Yucatan | Cultivated | INIFAP | 1.3 |
| ZEA4 | Criollo | Venezuela | Merida | Cultivated | FONAIAP | 5.0 |
